# Genotype Characteristics of *Giardia duodenalis* in Patients Using High Resolution Melting Analysis Technique in Khorramabad, Iran

**Published:** 2020

**Authors:** Akram SEPAHVAND, Ahmad HOSSEINI-SAFA, Hossein Ali YOUSOFI, Mohammad Hassan TAJEDINI, Reza PAHLAVAN GHAREHBABAH, Nader PESTEHCHIAN

**Affiliations:** 1. Infectious Diseases and Tropical Medicine Research Center, Isfahan University of Medical Sciences, Isfahan, Iran; 2. Department of Medical Parasitology and Mycology, School of Medicine, Isfahan University of Medical Sciences, Isfahan, Iran; 3. Department of Medical Parasitology and Mycology, School of Public Health, Tehran University of Medical Sciences, Tehran, Iran; 4. Applied Physiology Research Center, Isfahan University of Medical Sciences, Isfahan, Iran; 5. Department of Medical Biotechnology, Faculty of Advance Medical Science, Tabriz University of Medical Sciences, Tabriz, Iran

**Keywords:** *Giardia duodenalis*, Genotyping, Triosephosphate isomerase gene, High resolution melting, Iran

## Abstract

**Background:**

We aimed at genotyping and evaluating the predominance of *G. duodenalis* assemblages isolated from patients referred to medical laboratories in Khorramabad, Iran from Nov 2015 to Sep 2016. Hence, the development of a cost-effective HRM approach to determine genotypes of *G. duodenalis* based on the triosephosphate isomerase (tpi) gene was examined and the genotyping results with and without diarrhea was compared.

**Methods:**

Seventy *G. duodenalis* positive fecal samples were collected. A microscopic confirmation for the presence of *Giardia* spp. was performed, cysts of 70 *Giardia* spp. positive specimens were concentrated using sucrose flotation technique and sucrose solution PCR amplification was performed on 69 of 70 (98.5%) samples, and High Resolution Melting (HRM) analysis was performed using a software.

**Results:**

The results showed two distinct genotypes (assemblages A and B) of *G. duodenalis* but infections with mixture of both assemblages were not detected. The genotypes of *G. duodenalis* showed that the sub assemblage AI, BIII and BIV were present in a proportion of 68.1%, 20.3% and 11.6% respectively in samples. Assemblage AI was significantly (*P*<0.05) more frequently found in patients with diarrhea.

**Conclusion:**

The sub-assemblage AI, BIII, and BIV are more zoonotic potential. According to the comparison of the results of this study with the results of previous studies in this area and around of it, as well as the way people live and keep pets. This pattern established in Khorramabad city. HRM can be an ideal technique to detect and genotyping of *G. duodenalis* in clinical samples.

## Introduction

Giardiasis is the most common intestinal parasitic disease in Iran; in 10.9% of the general population (1). *Giardia* is a protozoan parasite that infects a wide range of vertebrate including humans. Among the six *Giardian* species such as *G. agilis*, *G. muris*, *G. microti* and *G. duodenalis*, only *G. duodenalis* has been found in human and a wide variety of other mammals (2, 3).”*G. duodenalis* is also of significant clinical importance in livestock (4, 5). “Giardiasis represents a public health concern in both developing and developed countries” (6, 7). *G. duodenalis* is a parasite in humans, and other vertebrates (8). Direct transmission can occur from infected people and animals to healthy individuals by direct contact; indirect transmission can occur through the ingestion of contaminated water (9, 10). *G. duodenalis* is still epidemic in developing countries such as Iran (1). 10.9 % of the study population. The prevalence of giardiasis in Iran is reported by 2%–36% (11). Due to the high prevalence of *G. duodenalis*, it is important to determine its genotypes and the source of the infection (3, 12).

According to the molecular characterization of nucleotide sequences of Glutamate Dehydrogenase (GDH), Elongation factor 1α (ef-1), Triosephosphate isomerase (tpi) and small subunit rRNA (SSU-rRNA) *gene* suggest the presence of eight assemblages (A–H) of *G. duodenalis* among which assemblages A and B are associated with human and a wide range of hosts infection while assemblages C, D, E, F and G,H are animal host-specific (3, 13–15); assemblage C, D, E, and F have also been isolated from infected humans (16). The assemblage A is divided into two subgenotypes AI and AII, whereas assemblage B includes BIII and BIV subgenotypes (17, 18). In the past years, sub assemblage within assemblage A and B (AI, AII, BIII, and BIV) have mainly been identified based on isoenzyme analysis; however, in this decade, genetic characterization has been extensively used to evaluate the genetic variability within *G. duodenalis* (14, 19).

molecular techniques such as multiplex-PCR (20), RFLP-PCR (12, 21, 22), Real-time PCR (23), RAPD-PCR (24) are used to molecular identification of *G. duodenalis* isolate, but in contrast to traditional melting analysis, the information in High Resolution Melting (HRM) can be analyzed based on the melting temperature (Tm) or the shape of the melting curve (25). HRM analysis based on the melting behavior with increasing temperature can determine “double-strand (dsDNA) DNA change to single-strand DNA (ssDNA)” (26). PCR products melting temperature is related to the sequence, length, CG percentage, or strand complementarily and the high-precision melting of PCR products makes it possible to discriminate samples which could provide sample differentiating down to single base-pair changes. (25). To gene scan by using HRM analysis, the shape of the melting transition of PCR products and temperature melting are important, respectively (27). Formerly, complete gene analysis was carried out by direct sequencing, scanning followed by sequencing and scanning followed by genotyping. Currently, rapid diagnosis and genetic analysis of pathogens demand increased concentration on evaluation and development mutation scanning methods. Data analysis is performed instead of several times electrophoresis by observing the melting curve of the PCR technique (26, 28, 29). Moreover, this method significantly decreases the test time and increases the efficacy of small amplified mutation detection (30). “Genotyping of some bacteria, viruses and a few parasites such as *G. lamblia* was performed by HRM” (25, 31).

The data on the genetic diversity of parasites in Iran is limited, thus, conducting such studies is necessary. The present study aimed at determining the distribution and predominance of *G. duodenalis* genotypes based on multi-locus analysis of the TPI gene. The development of a cost-effective HRM approach using the EvaGreen dye to identify genotypes of *G. duodenalis* human isolates and determining the frequency of different *G. duodenalis* assemblages among patients with giardiasis and comparing genotyping results with and without diarrhea from patients referring to medical laboratories at Khorramabad City was carried out.

## Materials and Methods

### Sample collection

Seventy *Giardia*-positive stool samples were collected from 491 people with intestinal disorders attending the primary health care centers who referred to medical laboratories of Khorramabad City, the west of Iran, from Nov 2015 to Sep 2016. Three fecal samples were collected from each person for stool exam.

Approval of Ethics Committee of all patients participating in the study were obtained IR.Iums.REC.393111. The samples were transferred to the Intestinal Parasites Researcher Laboratory in Department of Parasitology and Mycology, Faculty of Medicine, Isfahan University of Medical Sciences.

### Parasitological Examination

Microscopic confirmation was performed by examination on direct wet mount and stool concentration methods. All samples were confirmed positive for *Giardia* spp. cysts (8–10 cysts Ina 400X magnification field). Cysts of 70 *Giardia* spp. positive specimens were concentrated using four-layered discontinuous sucrose flotation technique (0.5, 0.75, 1, and 1.5 M) and single-layered sucrose solution (0.85 M) and (specific gravity 1.27) (32). The specimens with a high number of *Giardia* spp. cysts (100,000 in 1 ml) were kept at −20 °C until DNA extraction (12).

### Molecular characterization

#### DNA extraction

According to previous study for evaluation of four methods for extracting DNA from *G. duodenalis* cysts for PCR targeting the TPI gene, before DNA extraction, concentrated samples (200μL) were mixed with 200 mg crushed cover glass (0.4–0.5 mm) and vortexed for 1 min. Then the samples were boiled at 100 °C for 3 min) followed by freeze-thaw cycles and a 100 °C heating block (for six steps-each steps for 3 min) (33). Genomic DNA of all *G. duodenalis* positive fecal samples was extracted using genomic DNA extraction kit (Gene All, Seoul, Korea). Finally, the optical density (OD) and concentration of extracted DNA were measured at the wavelengths of 260 and 280 nm by Nano Drop device (WPA, England), and stored −20 °C.

### PCR amplification and HRM analysis

Genomic DNA was detected using specific primer for TPI gene, forward primer (CTTCATCGGYGGTAACTT) and reverse primer (TTCTGYGCTGCTATYYTC) were used (3). Amplification of DNA and HRM analysis was performed in 76 well plates on the Rotor-Gene 6000 (Hilden, Germany). The final reaction volume was 25μL. (Type-it HRM PCR Kit, Qiagen). Cycling reaction protocol was as follow: initial denaturation at 95 °C for 2 min, followed by 40 cycles of denaturation at 95 °C for 30 sec, annealing 55 °C for 27 sec and 72 °C for 20 sec followed by a final extension at 72 °C for 5 min. Fluorescence data were measured at the end of each cycle. After amplification, HRM analysis was performed from 80 to 90 °C at interval (ramps) of 0.2 °C increments passing for 2 sec per step for TPI gene (34, 35).

HRM analysis was performed by using Rotor–Gene 6000 series software version 1.7 (Corbett, Hilden, Germany). For analysis, initial data were normalized by applying curve fitting scale for best line. So highest fluorescence value equalized as 100 and lowest equalized as zero. Then, the curves were differentiated. The median curves were composited using the median fluorescence of all samples. Finally, the difference in melting curve shapes and Tm points were used to determine the sample genotypes. Tm analysis was repeated triplicate in each run to confirm the reproducibility of the Tm assay by calculating the Tm variation within and between PCR amplification run.

### DNA Sequencing and Phylogenetic analysis

Eighteen samples were sequenced to confirm the detection of genotypes and correlating theses with HRM results. Representative nucleotide sequences of *G. duodenalis* were deposited in GenBank under accession numbers I02120 for AI, U57897 for AII, AF069561 for BIII and AY368163 for BIV. The subtypes of *G. duodenalis* identified in this study were analyzed with known ones using a maximum Likelihood analysis of the aligned sequence implemented in the program Mega 6 (36).

### Statistical analysis

Patients with giardiasis were grouped by diarrheic or non-diarrheic to determine whether these factors were associated with G. duodenalis assemblages, using Chi-square analysis, Fisher’s exact test, and Z test. Statistical comparisons were carried out using SPSS 16.0 statistical software (Chicago, IL, USA). Differences were considered significant when (*P*<0.05).

## Results

Only 70 people stool samples confirmed *Giardia* infection. Cysts and trophozoites were observed in all fecal isolates after direct and concentration methods and staining by Lugol’s iodine. Out of 70 giardiasis cases, 47/70 were with diarrheal and 23/70 without diarrheal (12).

DNA from stool samples of 70 patients was extracted. The molecular survey and PCR amplification were successfully performed on 69 from 70 (98.5%) *G. duodenalis* samples. In our survey, the genotypes of *G. duodenalis* showed that the sub-assemblage AI, BIII and BIV were present in a proportion of 68.1%, 20.2%, and 11.5% respectively in samples and mixed assemblages were not found in the study. Assemblage AI was significantly (*P*<0.05) more frequently found in patients with diarrhea on 40 from 69 patients, assemblages A frequency in patients suffering from diarrhea was almost 5.7 times higher than B assemblages; 22 (31.8%) patients were free of diarrhea (Table 1). A mixture of sub-assemblages has not been recorded in the isolates from the present study.

**Table 1:** Frequency of assemblage A and B in patients with giardiasis base on diarrhea

***G. duodenalis Assemblage***	***Total No. (%)***	***Diarrheal Patients (%)***	***Non-Diarrheal Patients (%)***
AI	47(68.1)	40(57.1)	7(10.1)
BIII	14(20.2)	4(5.8)	10(14.5)
BIV	8 (11.6)	3(4.3)	5(7.2)
Total	69(100)	47(68.1)	22(31.8)

*P*-value<0.05 indicates significant association

**Fig. 1:**
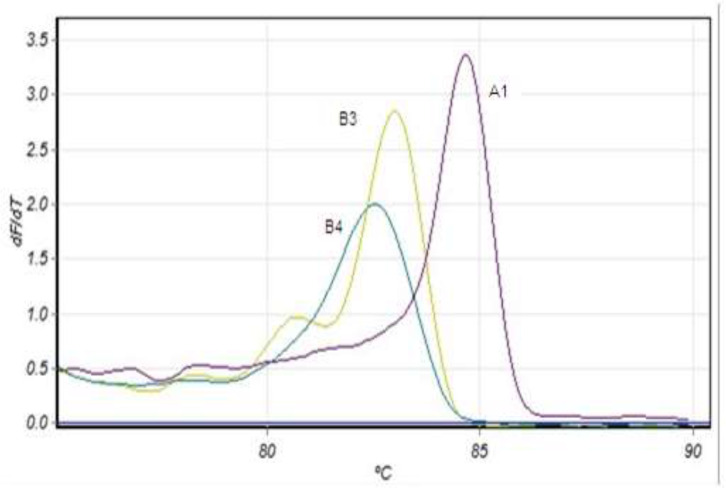
First derivation of the fluorescence (^L^d*F*/d*T*) versus temperature with manually assigned samples to the corresponding assemblage identified by sequencing A1: genotype AI. B3: genotype BIII. B4: genotype BIV

HRM analysis of amplification dependents on DNA melting is illustrated in (Fig. 1). The melting temperature of the peaks varied between and within runs; the relationship between three peaks observed for assemblage AI, BIII and BIV showed little variation. Assemblage AI showed a peak between 84.2 and 84.7 °C assemblage BIII a peak between 83 and 83.8 °C and assemblage BIV a peak between 82.2 and 82.9 °C (Fig. 1 and Table 2).

**Table 2:** Tm analysis and differentiation between *G. duodenalis* genotypes

***Assemblage***		***AI***	***BIII***	***BIV***
N		47	14	8
Mean		84.5	83.3	82.6
SEM		0.01	0.06	0.06
SD		0.12	0.24	0.2
CV%		0.14%	0.2%	0.24%
CI95%	Lower	84.4	83.1	82.4
	Upper	84.5	83.4	82.7
Minimum		84.2	83	82.2
Maximum		84.7	83.8	82.9
Total		69	69	69
percent		68.1%	20.2%	11.6%

AI: Genotype AI. BIII: Genotype BIII. BIV: Assemblage BIV.CV: coefficient of variation. CI: Confidence interval. N: number. SD: standard deviation. SEM: standard error of the mean

**Fig. 2:**
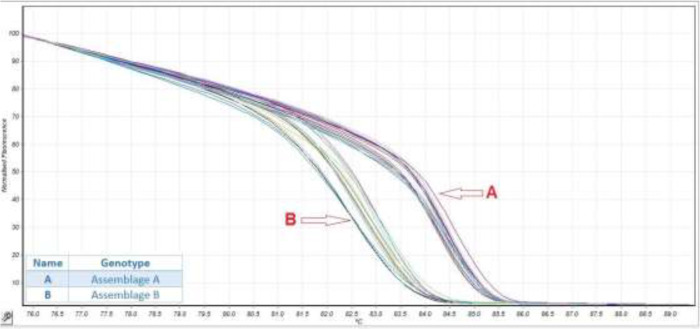
Principle of high-resolution amplification melting curve analysis to product fluorescence different plot curves

**Fig. 3:**
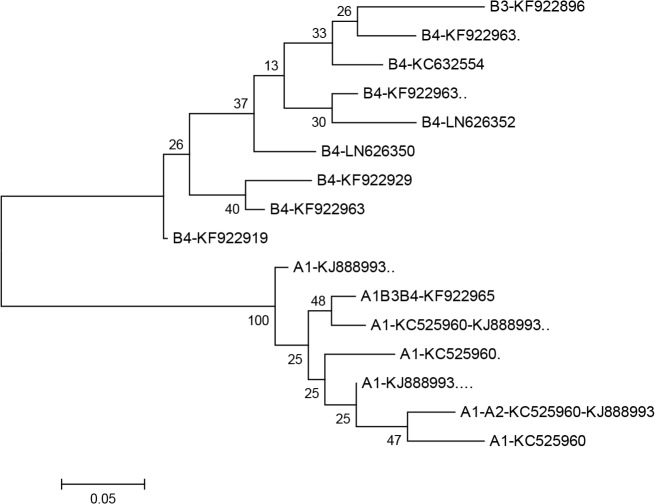
Phylogenetic relationships of *Giardia duodenalis* inferred by the maximum Likelihood analysis on concatenated *tpi* gene nucleotide sequences, implemented in MEGA version 6.0. A1: Genotype AI. B3: Genotype BIII. B4: Genotype BIV

For better discrimination of small sequence variations among AI, BIII and BIV, due to the short amplification, we normalized raw data by applying curve scaling to alien of best fit from the standard normalized melt curve (Fig. 2). To confirm the accuracy of the HRM assay, we sequenced 18 samples. The results were correctly concordance with HRM assay. The phylogenetic tree was divided into two main clades: the first clades contained two subclades corresponding to the BIII and BIV genotypes, and the second one corresponding to the AI genotypes (Fig. 3).

## Discussion

This study aimed to evaluate and genotyping of *G. duodenalis* isolated from infected humans with and without diarrhea in Khorramabad city using the HRM technique. HRM can be an ideal, sensitive, satiable and accurate technique for detection and genotype of *G. duodenalis* in clinical samples. Furthermore, a cost-effective HRM approach using Eva Green (EG) dye to determine genotypes of *G. duodenalis* based on the triosephosphate isomerase gene was described. Compared to these methods, HRM-based methods, as completely “closed-tube” formats, are attractive because they could offer several methodological advantages (such as rapid turnaround time, no post-PCR processing steps) over other conventional gene scanning methods.

Regarding the use of HRM for genotyping in some parasitic infections, samples with single peaks indicate homozygous genotype and those with two peaks indicate heterozygous genotypes (27). In this study, the results of DNA sequencing confirm HRM assay, and the results were in concordance with HRM assay.

Several researchers (3, 37) highlighted some advantages of HRM-based methods, but in Iran, no study could be found based on HRM method for detection and genotyping of *G. duodenalis*. More research is recommended to investigate the prevalence and situation of giardiasis in humans and animals.

It is known that *G. duodenalis* is a species complex. It is shown that the distribution of two assemblages among human-associated *Giardia* isolates varies in different parts of the world. In America, there seem to be pockets of areas with differing predominant assemblages. Studies done in Mexico, Brazil and Colombia identified higher frequencies of assemblage A. Sub-assemblage AI had been the predominant sub-assemblage A identified, while studies from Nicaragua and Argentina show that assemblage B is predominant (20). As Meloni “assemblage B isolates appear to be less widespread and restricted to localized endemic foci” (38). The distribution of BIII and BIV infections in humans varied depending on the geographic region and environmental factors. BIII was predominantly detected in Africa, Asia, the Middle East, as well as Central and South America, whereas BIV was more likely to be detected in North America. The frequencies of BIII and BIV were not much different in Australia and Europe (14). Similar to all studies, depending on the geographical area and environmental factors, the sub-assemblages AII, BIII, and BIV were found in Khorramabad; however, the frequency was different compared to other parts of Iran. Similar to other parts of the world, in Iran, the frequency of type sub-assemblages AII and BIII are reported to be higher than other assemblages (11).

Sub-assemblages AI and AII are found in both animals and humans. Sub-assemblage AI primarily detected in pets and livestock is most often responsible for zoonotic transmission, whereas sub-assemblage AII is predominantly found in humans (12, 19, 22). AI sub-assemblage and sub-assemblages BIII and BIV had a broad host range including pets and livestock. Assemblages BIII and BIV can be found in both humans and animals and “appear to be largely human-specific, it has been reported in some animals and may represent a zoonotic potential” (2, 23). This is consistent with a zoonotic origin of infection that could be due to the contamination of public water with raw sewage from animal and human sources (39). Our findings, in a similar vein, revealed that most AI sub-assemblage was largely correlated with the maintenance of pets, livestock and keeping cats and dogs as well as stray dogs and environmental factors such as not having a sewage network in the area and geographic conditions, culture, and climate. Similar results were found in another study in Shahre-Kord (40).

Studies on 30 patients based on the (gdh) gene, the positive rate of *G. duodenalis* was 24 fecal samples. The findings are in line with the results of our study which showed that all samples were only genotype B (although in the above study, the sub-assemblages were not examined to show, more accurately, the zoonotic transmission of the disease in the area (41).

In another study (42) in Kurdistan Province, “assemblages A and B were the most common types of human giardiasis and giardiasis could be a zoonosis disease”.

The zoonotic origin of the giardiasis was prominent suggesting that most of the infections were only zoonotic origins.

However, the results of the present study do not correspond with the findings of some other reports such as in England (43). *G. dudenalis* was detected in humans mostly belonged to assemblage B (44). In Iran, sub-assemblages AII had higher frequency followed BIII and BIV which is different from our study (10, 12, 17, 21, 45); the results of the present study established the zoonotic transmission pattern in Khorramabad City. According to HRM genotyping, sequencing and phylogenetic analysis suggested AI as the most common assemblages and zoonotic transmission.

There is also a relationship between assemblages and symptoms (diarrhea and fever). In this research by comparing the frequency of assemblage A 40(57.1) and Assemblage B 7(10.1), we found that Assemblage AI was significantly (*P*<0.05) more frequently found in patients with diarrhea on 40 from 69 (57.1%); A assemblages frequency in patients suffering from diarrhea was almost 5.7 times higher than B assemblages. We found a correlation between assemblage A and symptomatic infections, and between assemblage B and asymptomatic infections in all the patients. In most areas, assemblage A was in association with diarrhea and fever these results agree with them (44–46).

Considering the variability of assemblages, it seems necessary to further research especially multi-locus gene and examine the relationships among new possible sources and reservoirs.

## Conclusion

The sub-assemblage AI, BIII, and BIV are more zoonotic potential. According to the comparison of the results of this study with results of previous studies in this area and around of it, as well as the way people live and keep pets. Thus, this pattern established in Khorramabad city. HRM can be an ideal, technique to detect and genotyping of *G. duodenalis*.
